# Activity of pembrolizumab in relapsed/refractory NK/T-cell lymphoma

**DOI:** 10.1186/s13045-018-0559-7

**Published:** 2018-01-31

**Authors:** Xin Li, Yasong Cheng, Mingzhi Zhang, Jiaqin Yan, Ling Li, Xiaorui Fu, Xudong Zhang, Yu Chang, Zhenchang Sun, Hui Yu, Lei Zhang, Xinhua Wang, Jingjing Wu, Zhaoming Li, Feifei Nan, Li Tian, Wencai Li, Ken H. Young

**Affiliations:** 1 Department of Oncology, Zhengzhou University First Affiliated Hospital; Lymphoma Diagnosis and Treatment Center of Henan Province, Νo. 1 Jianshe East Road, Zhengzhou, Henan China; 2 Department of Pathology, Zhengzhou University First Affiliated Hospital, Νo. 1 Jianshe East Road, Zhengzhou, Henan China; 3 Department of Hematopathology, The University of Texas MD Anderson Cancer Center, Houston, TX USA

**Keywords:** NK/T-cell lymphoma, PD-1 blockade, Pembrolizumab

## Abstract

Natural killer/T-cell lymphoma (NKTCL) is a rare subtype of non-Hodgkin lymphoma that is associated with a poor outcome. Currently, the treatment needs of NKTCL remain unmet, and efforts to further improve treatment are urgently needed. Herein, seven patients with NKTCL who failed to respond to various types of chemotherapies were treated with the anti-programmed death 1 (anti-PD-1) antibody pembrolizumab at 100 mg every 3 weeks. After a median of four cycles of treatment (range 2–18), four out of seven patients responded (two complete response, two partial response, overall response rate 57%). Expression of PD1-ligand available was 50, 20, 30, 70, and 30% of five patients respectively. It is negative in one patient and not tested in one patient. Adverse events, which mostly ranged from grade I to grade III, were tolerable and could be safely handled, although immune-related pneumonitis was notable. Overall, PD-1 blockade with pembrolizumab represents a favorable strategy for the treatment of refractory/relapsed NKTCL.

## Background

Immunotherapeutics, specifically immune checkpoint inhibitors of the PD-1 (programmed death 1)/PD-L1 (programmed death ligand 1) pathway, is an extremely active area of laboratory and clinical investigation [1] and has demonstrated utility as targets in advanced cancer, with evidence of both an overall survival benefit and durable responses [2, 3]. Many clinical trials of PD-1 blockade treatment in solid tumors and hematological tumors (including malignant lymphoma) have been conducted [4–6]. Pembrolizumab and Nivolumab have been approved for use in multiple types of cancer, including melanoma, non-small cell lung cancer, renal cell carcinoma and squamous cell carcinoma, which has led to unprecedented clinical progress [7–9].

The administration of immune checkpoint inhibitors in hematological tumors, especially classic Hodgkin lymphoma, has developed fast these years [10]. Classical HL proves to be a promising target for anti-PD-1 therapy because PD-L1 is overexpressed by Reed-Sternberg cells [11] and PD-1 blockade Nivolumab has been tested in many clinical trials and obtained favorable results [12, 13]. It was also evaluated in a cohort of patients with relapsed or refractory lymphoid malignancies, including 29 with B-NHL, 2 with PMBCL, and 23 with T-NHL. Four (36%) patients with DLBCL, four (40%) with FL, two (15%) with mycosis fungoides, and two (40%) with peripheral T cell lymphoma responded to the therapy, among whom one patient (9%) with DBLCL and one (10%) with FL achieved CR [14, 15]. In general, non-Hodgkin lymphomas (NHLs) do not share cHL’s vulnerability to PD-1 inhibitors, and the majority of NHLs appear to be minimally sensitive to PD-1 blockade [1].

NK/T cell lymphoma, which has a distinctive morphology, immunophenotype, and biological behavior, relapses frequently and progresses rapidly. However, there are few investigations into relapsed/refractory NK/T cell lymphoma and no standard treatments yet available. Only sporadic studies about PD-1/PD-L1 blockade treating NK/T cell lymphoma have been reported [16].

In our retrospective study, a total of seven patients with refractory/relapsed NKTCL were treated with pembrolizumab at our lymphoma diagnosis and treatment center. Herein, we report our findings of PD-1 blockade with pembrolizumab in these highly refractory NKTCL patients.

## Patients and methods

### Patients and treatment

A total of seven patients with refractory/relapsed NKTCL were treated with the anti-PD1 antibody pembrolizumab. All patients had received at least two prior chemotherapy regimens. Pembrolizumab at 100 mg was administered every 3 weeks in all patients. All patients were fully informed about the nature and possible toxicities of the treatment protocol and gave informed consent.

### Response assessments and monitoring

A computed tomography (CT) scan with contrast and/or fluorodeoxyglucose (FDG) positron emission tomography/computed tomography (PET/CT) were used to assess treatment responses according to the Revised Response Criteria for Malignant Lymphoma. Circulating EBV (Epstein Barr virus) DNA and lactate dehydrogenase (LDH) levels were measured. Adverse events (AEs) were graded according to the National Cancer Institute Common Terminology Criteria for Adverse Events, version 4.0.

## Results

### Patients

A total of seven patients with refractory/relapsed NKTCL were enrolled. All seven patients (median age: 47, range 17–61 years) had one to two of Eastern Cooperative Oncology Group performance status (ECOG PS). The median number of prior treatment regimens was four (range 3–10). The patient characteristics are shown in Table 1.

**Table 1:** Patient characteristics

Case	Sex	Age (years)	Bone marrow	Stage	PINK score		Sites	Prior treatment
1	F	39	Negative	IV	2		Nasal cavities, skin of upper and lower limbs, cervical, axillary and inguinal lymph nodes	DDP+VP16+IFO(2); pegaspargase+MTX+ examethasone (2); liposomal doxorubicin+gemcitabine+pegaspargase+dexamethasone(3)
2	M	31	Negative	II	3		Mediastinal, hilar, para-aortic, mesenteric, right iliac vessels lymph nodes, intestinal, transverse colon and rectum	pegaspargase+MTX+dexamethasone(4); hydroxycamptothecin+paclitaxel+mitoxantrone+methylprednisolone(6); Auto-HSCT
3	F	61	Negative	II	4		Left cervical, inguinal lymph nodes, left upper arm, right forearm, buttock, subcutaneous soft tissue of left lower leg, left lung upper lobe, and right lung middle lobe	CTX+VCR+adriamycin+prednisone(8); pegaspargase +DDP+gemcitabine+ dexamethasone(4)
4	M	53	Negative	II	1		Sinus	DICE+L-Asp(4); CHOP+L-Asp(3)
5	M	61	Negative	IV	3		Left posterior lateral wall of the oropharynx, left cervical lymph nodes, right lung, spleen, and adrenal gland	GDPT(6); DDGP(2)
6	M	47	Negative	IIIEB	3		Parotid gland, oropharynx, nasopharynx, spleen, cervical, left supraclavicular, hilar, mediastinal, and inguinal lymph nodes	DICE+L-Asp(2); DICE+pegaspargase(2)
7	F	17	Negative	IEB	0		Left nasal cavity, chest wall	VIPD(3); DDGP(4)

The baseline characteristics of seven cases, including gender, age, organ involvement, and main previous treatment regimens

*DDP*, cisplatin; *VP16*, etoposide; *IFO*, ifosfamide; *MTX*, methotrexate; *Auto-HSCT*, autologous hematopoietic stem cell transplantation; *CTX*, cyclophosphamide; *VCR*, vincristine; *DICE*, dexamethasone, ifosfamide, cisplatin and etoposide; *L-Asp*, L-asparaginase; *CHOP*, cyclophosphamide, epirubicin, vincristine and prednisone; *GDPT*, gemcitabine, cisplatin, dexamethasone and thalidomide; *DDGP*, cisplatin, dexamethasone, gemcitabine and pegaspargase; *VIPD*, cisplatin, etoposide, ifosfamide and dexamethasone

### Clinical outcomes

A median of four (range, 2–18) cycles of pembrolizumab was administered. The overall response rate (ORR) was 57.1% (95% confidence interval [CI], 18 to 90%), with a complete response (CR) occurring in two (28.6%) patients and a partial response (PR) observed in two (28.6%) patients. Response duration, PFS, and OS of seven patients are 4.1 months, 4.8 months, and 5.0 months respectively.

**Fig. 1:**
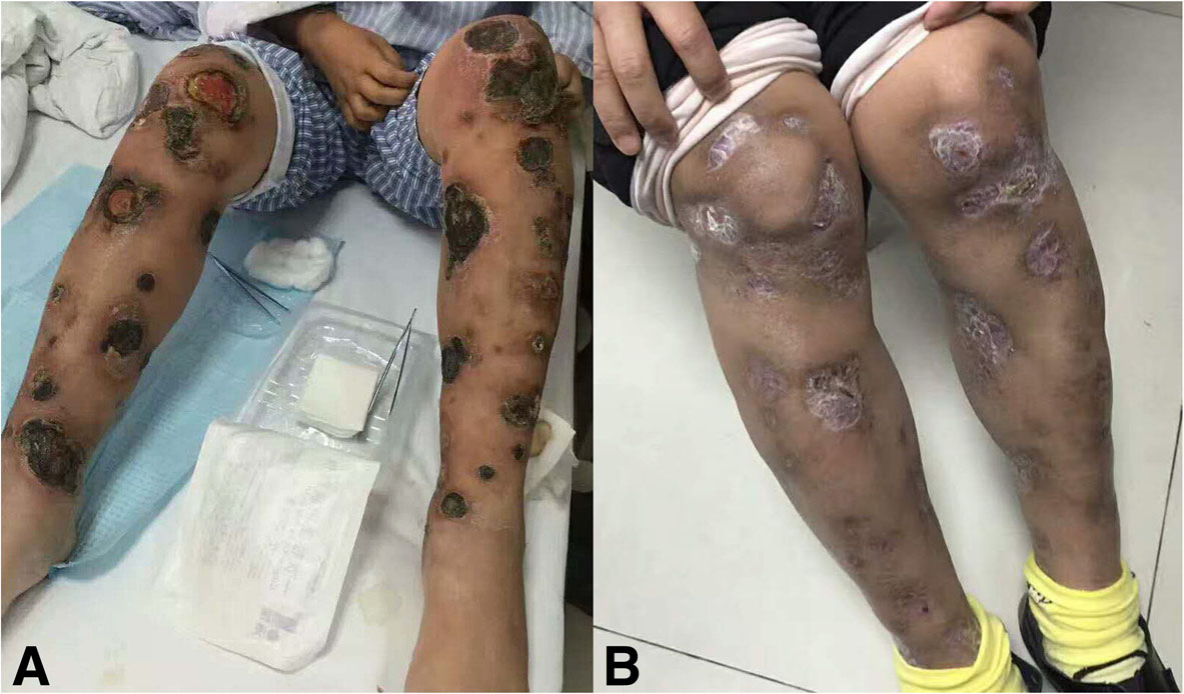
Lesion changes of case 1 before and after pembrolizumab treatment. **a** The skin lesions of lower limbs of case 1 at the time of relapse. **b** The skin lesions responded after the first cycle. After 4 cycles, her crust of the lesions fell off and ulcers healed

Case 1 had biopsy-confirmed extensive skin involvement. Pembrolizumab treatment led to rapid improvement of skin lesions, and PET/CT scans after 4 cycles showed a metabolic CR. The skin lesions in this patient’s lower limbs disappeared (Fig. 1) and were replaced by granulation tissue 5 cycles later. EBV DNA level was measured before treatment. At the time of this report, this patient has completed 18 cycles of pembrolizumab treatment.

**Fig. 2:**
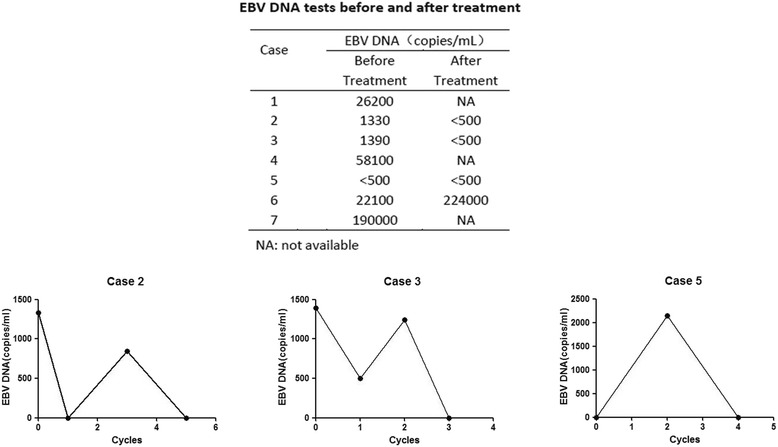
Changes in circulating EBV DNA with pembrolizumab treatment. The EBV DNA levels in case 2 fell from 1330 copies/ml to < 500 copies/ml after 5 cycles. The EBV DNA levels in case 3 fell from 1390 copies/ml to < 500 copies/ml after 3 cycles. The EBV DNA levels in case 5 rose from normality to 2140 copies/ml after 2 cycles and went back to normal 4 cycles later. The EBV DNA levels of case 6 rose from 22,100 copies/ml to 224,000 copies/ml after 3 cycles. The EBV DNA levels of case 7 rose gradually from 10,900 copies/ml to 190,000 copies/ml prior to pembrolizumab treatment

**Fig. 3:**
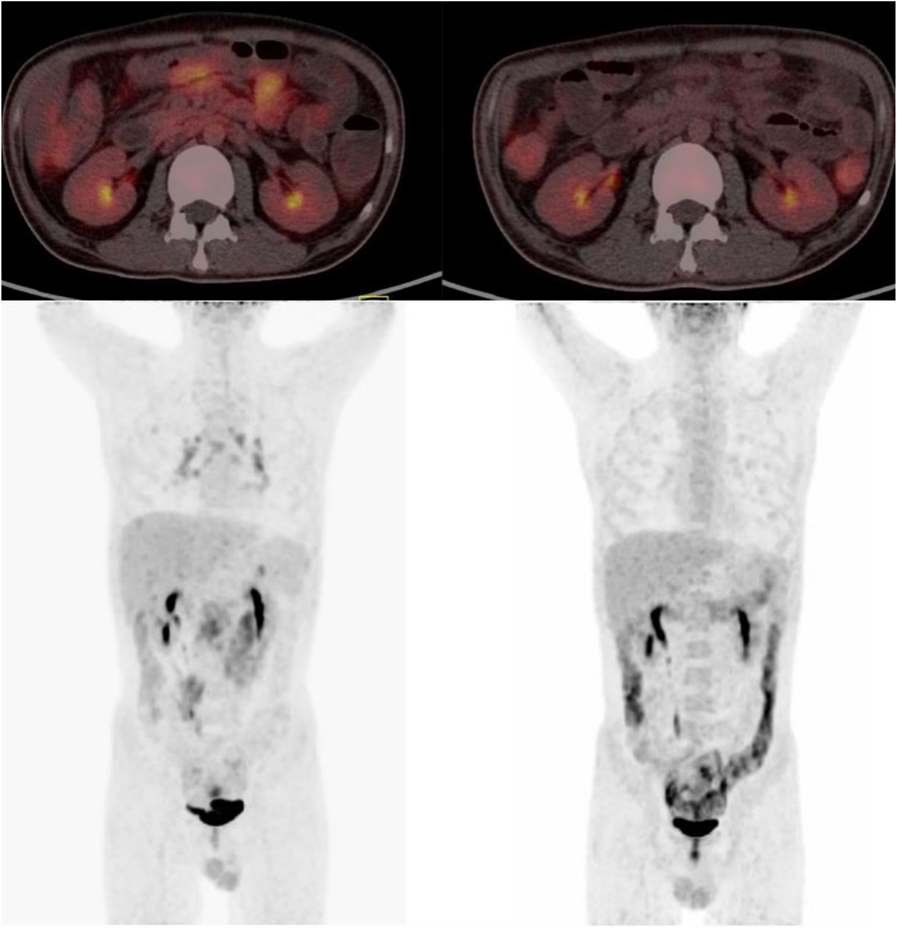
PET/CT results of case 2. The scan of case 2 in the left two images showed a relatively hypermetabolic lesion in mediastinal, hilar lymph nodes, and intestines after using pembrolizumab for 1 cycle. The two images on the right showed that the lesions were metabolically less active 2 cycles later

The EBV DNA levels in case 2 (Fig. 2) fell from 1330 copies/ml to < 500 copies/ml after 5 cycles, and the LDH levels fell from 298 U/L to 147 U/L. A PET/CT scan showed a metabolic CR after 2 cycles (Fig. 3).

The EBV DNA levels in case 3 (Fig. 2) fell from 1390 copies/ml to < 500 copies/ml after 3 cycles. CT scans in cases 3 and 5 showed PR status.

Case 4 developed symptoms of dyspnea and low oxygen saturation. Consequently, pembrolizumab was discontinued. The EBV DNA levels in case 5 (Fig. 2) rose from normality to 2140 copies/ml after 2 cycles and went back to normal 4 cycles later. The EBV DNA levels of case 6 (Fig. 2) rose from 22,100 copies/ml to 224,000 copies/ml after 3 cycles, and this patient ultimately developed hemophagocytic syndrome (HPS). The symptoms of case 6 did not abate after the application of anti-HPS therapy, and the patient died of progressive disease (PD). The EBV DNA levels of case 7 (Fig. 2) rose gradually from 10,900 copies/ml to 190,000 copies/ml prior to pembrolizumab treatment.

### PD-L1 expression on lymphoma cells

**Fig. 4:**
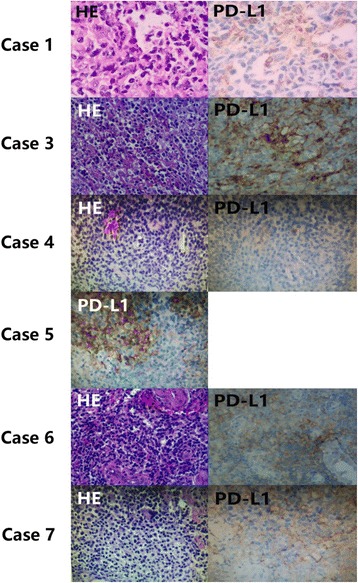
HE staining and IHC of six patients available. The scan of case 2 in the left two images showed a relatively hypermetabolic lesion in mediastinal, hilar lymph nodes, and intestines after using pembrolizumab for 1 cycle. The two images on the right showed that the lesions were metabolically less active 2 cycles later

PD-L1 expression was measured by immunochemistry on formalin-fixed, paraffin-embedded tissue sections (Fig. 4). The expression percentage of PD-L1 expressed on lymphoma cells of case 1, case 3, case 5, case 6, and case 7 is 50, 20, 30, 70, and 30% respectively (Table 2). PD-L1 expression data were not available for case 2. We haven’t observed direct connection between PD-L1 expression and clinical response.

**Table 2:** The expression of PD-L1, CD3, CD4, CD8, and EBER

Case	PD-L1	CD3	CD4	CD8	EBER
1	50%	+	NA	NA	+
2	NA	+	−	+	+
3	20%+	+	NA	NA	+
4	−	+	NA	NA	NA
5	30%+	+	+	NA	+
6	70%+	NA	+	+	+
7	30%+	+	NA	NA	+

The expression of PD-L1, CD3, CD4, CD8, and EBER on lymphoma cells. PD-L1: It is not available for case 2 and negative for case 4. The expression ratio of the other five cases is 50, 20, 30, 70, and 30% respectively. Most cases have positive CD3 expression except case 6. EBER is not available for case 4 and the remaining six cases are positive. More than half of the cases didn’t receive CD4 and CD8 tests

*NA*, not available

### Adverse events

Treatment-related AEs of any grade occurred in 71.4% of patients. The main AEs were pneumonitis and laboratory abnormalities. Cases 2 and 6 suffered from diarrhea. Case 3 experienced a fever. Case 4 developed grade 3 thrombocytopenia. The patient was treated with thrombocyte transfusion and recombinant human thrombopoietin, and the thrombocyte count climbed to normality. Cases 4 and 7 presented with grade 3 pneumonitis. Both of them suffered from respiratory failure and did not respond well to corticosteroid and advanced respiratory support treatment. The other patients experienced no treatment-related AEs (Table 3).

**Table 3:** Adverse events possibly related to pembrolizumab

Event	Grade 1	Grade 2	Grade 3	Total *n* = 7(%)
Pneumonitis	0	0	2	2 (28.6%)
Diarrhea	1	0	0	1 (14.3%)
Pyrexia	1	0	0	1 (14.3%)
Anemia	1	1	0	2 (28. 6%)
Neutropenia	1	1	0	2 (28. 6%)
Thrombocytopenia	1	0	1	2 (28. 6%)
Increased ALT	0	1	0	1 (14.3%)
Increased AST	2	0	0	2 (28. 6%)

Adverse events we encountered during the course of pembrolizumab treatment. Most adverse reactions belong to grade I to grade II. Two cases suffered from pneumonitis and one case developed thrombocytopenia

*ALT*, alanine aminotransferase; *AST*, aspartate aminotransferase

## Discussion

NK/T-cell lymphoma (NKTCL) is a subtype of non-Hodgkin lymphoma that is more prevalent in China than in Western countries [17, 18]. Moreover, traditional treatments offer especially poor efficacy and prognoses [19, 20]. Studies have shown that NKTCL cells play an important role in the activation and tolerance of T cells, as these cells can avoid immune surveillance and the consequent killing of NKTCL, resulting in a poor prognosis.

The consensus on standard treatment for NK/T cell lymphoma has not been reached until now. Current data indicate that advanced-stage and relapsed/refractory NK/T-cell lymphoma should be treated with L-asparaginase-containing regimens that incorporate non-MDR-dependent drugs [17, 21–23]. Additionally, HSCT has been explored in NK/T-cell lymphoma [24, 25]. Treatment options, especially targeted drugs for patients with relapsed/refractory NKTCL are limited. A retrospective study has shown that the estimated 5-year OS of 47 patients undergoing autologous HSCT was 56% [26]. In another retrospective analysis, three patients with NK/T-cell lymphoma undergoing allogeneic HSCT were studied, and the 3-year OS and PFS were 55 and 53% [27]. Continued efforts should be made to improve chemotherapeutic regimens and other targeted drugs.

Few studies have been conducted to explore the application of PD-1 blockade in the treatment of NKTCL. Therefore, large-scale clinical trials on pembrolizumab remain to be conducted to assess and confirm the treatment outcomes in NKTCL. The B7 family co-stimulatory molecule PD-L1 plays a key role in the activation and tolerance of T cells [28, 29]. PD-L1 interacts with its receptor, PD-1, and transmits a negative regulatory signal that induces tumor antigen-specific T-cell apoptosis and immune dysfunction and promotes immune escape of lymphoma cells [30, 31].

Programmed death receptor 1 (PD-1) is an inhibitory receptor expressed on the surface of activated T cells and is normally involved in immune tolerance and the prevention of tissue damage associated with chronic inflammation. Interactions of PD-1 with its ligands, PD-L1 and PD-L2, inhibit T-cell receptor signaling by downregulating T-cell activation and proliferation and blunting T-cell-mediated anti-tumor immune responses [32, 33]. Thus, the PD-1 pathway represents an immune checkpoint that acts to suppress anti-tumor immunity. Studies have shown that T-cell function and anti-tumor responses can be enhanced by anti-PD-1 and anti-PD-L1 antibodies in mouse models of various types of tumors [34–39].

In our study, the expression ratio of PD-L1 expressed on lymphoma cells of case 1, case 3, case 5, case 6, and case 7 is 50, 20, 30, 70, and 30% respectively. In case 4, there was no detected PD-L1 expression, and data were not available for one patient (case 2). We found that cases 1 and 3 achieved a CR and PR, respectively; case 5 achieved a PR. However, two patients (cases 6 and 7) had disease progression. We did not find out a direct relation between the expression of PD1-ligand and clinical response. Due to availability of the drug and financial limitations, our cases received lower dosages of pembrolizumab, which could have accounted for the difference in response rate. Additional factors may affect the responses to PD-1 blockade. One study found that PD-L1 expression was positively correlated with EBV-driven LMP1 (latent membrane protein 1) expression at both the protein and mRNA levels in NKTCL and NK cells [40]. Among three patients mentioned in another study, EBV DNA copy numbers were downregulated after pembrolizumab treatment, which might indicate that EBV infection acts as a possible mechanism for inducing PD-L1 expression [41]. Considering the relationship between PD-L1 expression and EBV activity [42], PD-1 blockade may play a significant part in restoring immunologic function and reducing EBV copy numbers. In addition, EBV copy numbers may serve as a predictive indicator of both the treatment outcome and prognosis of NKTCL. In this study, patients (cases 6 and 7) with higher post-treatment EBV level had worse response than that in patients with lower post-treatment EBV level. The study of Kwong et al. showed that in all clinical subtypes of NK cell lymphomas, EBV DNA was an important prognostic element for disease-free survival (DFS) and overall survival (OS) [43, 44].

One of the seven patients in the study developed mild pyrexia. Cytokine release and nonspecific activation of an immune response is postulated to account for the development of fever [45]. Hepatic AEs occurring after treatment with immune checkpoint inhibitors are mainly characterized by asymptomatic elevations in aspartate aminotransferase and alanine aminotransferase level [46]. In some large-scale clinical trials of anti-PD1 antibodies, the occurrence rate of hepatitis was under 5%, and grade 3 or 4 toxicity was rarely observed [47, 48]. Pneumonitis is generally defined as inflammation of the pulmonary parenchyma. In our study, both cases 4 and 7 suffered from serious pneumonitis. Accordingly, it is critical to initiate treatment of pneumonitis as soon as possible. In terms of treatment, corticosteroids remain fundamental for treating immune-related adverse events (irAEs) [49]. Case 2 experienced diarrhea, and considering that this patient was diagnosed with enteropathy-associated T-cell lymphoma, his symptoms were not solely derived from PD-1-related colitis. PD1/PD-L1 blockade therapies have been associated with a lower risk of hematologic toxicities [49]. Furthermore, cases 4 and 6 had previously been treated with various types of chemotherapies. Thus, we assumed that the hematologic toxicities of these two patients were primarily consequences of their prior chemotherapies. As immunotherapies become more common in the clinical management of patients with many different types of cancer, it is essential to understand irAEs. Moreover, tumor neoantigens and normal tissue antigens can be cross-reactive, leading to irAE generation during immunotherapy [50, 51]. Cytopenia is rarely associated with immune checkpoint blockade in patients with solid tumors but appears to occur more frequently in patients with lymphoma [52]. For cases 4 and 6, the unexplained elevation of serum levels of hepatic alanine aminotransferase and aspartate aminotransferase enzymes suggested the presence of immune-related hepatitis. In general, the majority of AEs related to pembrolizumab treatment were mild and controllable, mostly grade 1 or 2 in our study, demonstrating that pembrolizumab can be safely administered to patients with NKTCL.

## Conclusions

This retrospective study of seven patients with refractory NKTCL shows that pembrolizumab administered at doses of 100 mg every 3 weeks were effective. The relationship between PD-L1 expression and response to PD-1 blockade is inconclusive in this analysis because of small sample size. Further studies are warranted to evaluate and confirm the activity of PD-1 blockade in patients with NKTCL.

## Abbreviations

Anti-PD-1: Anti-programmed death 1
CI: Confidence interval
CR: Complete response
CT: Computed tomography
DFS: Disease-free survival
EBV: Epstein Barr virus
FDG: Fluorodeoxyglucose
irAEs: Immune-related adverse events
LDH: Lactate dehydrogenase
LMP1: Latent membrane protein 1
MAPK: Mitogen-activated protein kinase
ORR: Overall response rate
OS: Overall survival
PD-L1: Programmed death ligand 1
PET/CT: Positron emission tomography/computed tomography
PR: Partial response
